# Mental Insanity Assessment of Pedophilia: The Importance of the Trans-Disciplinary Approach. Reflections on Two Cases

**DOI:** 10.3389/fnins.2018.00335

**Published:** 2018-05-16

**Authors:** Cristina Scarpazza, Ambrogio Pennati, Giuseppe Sartori

**Affiliations:** ^1^Department of Psychology, University of Padua, Padova, Italy; ^2^Department of Psychosis Studies, King's College Health Partners, King's College London, London, United Kingdom; ^3^Associazione Scientifica Integrational Mind Labs, Milan, Italy

**Keywords:** neuroscience, mental insanity, complexity, social phenomena, acquired pedophilia

## Abstract

A 60 plus-year-old male was charged with pedophilia for forcing a child to touch him inappropriately near a primary school fence. In another case, a 70 plus-year-old male was charged with pedophilia for intimately touching a boy in a cinema. What led them to manifest this socially-inappropriate and legally-relevant behavior? Is there an explanation for the sexually-related behavioral changes emerging late in life of these two men? Indeed, a common point exists between the two men: both were found to suffer from highly-disabling neurological conditions, known to have a potential effect on social behavior. Specifically, a large right frontoparietal meningioma was found to have important influence on the first man's cognition and control inhibition, whereas frontotemporal dementia prevented the second man from understanding the moral disvalue of his sexually-inappropriate behavior and controlling his sexual impulses. In the current presentation, particular emphasis is placed on the logical reasoning supporting the conclusions that both the pedophiles should be considered not guilty by reason of insanity. Furthermore, experimental methods have been used to explore both cases, which rely on the existence of cognitive models for the phenomena under study, the integration of insights offered by different disciplines and the application of a variety of tools and approaches that follow the “convergence of evidence” principle, which could be safely used in court to support a mental insanity claim. Here, we describe how the use of the experimental method could become useful to reduce the uncertainty in mental insanity assessments. The use of a transdisciplinary, scientifically-grounded approach can help to change the way legal phenomena are interpreted. For instance, when assessing mental insanity, consultants should not only investigate the eventual existence of a diagnosis, but should assess the cognitive/affective abilities that are necessary to understand our own behavior and emotions as well as those of others. The criteria for responsibility should be symptoms-based and not diagnosis-based. Since pedophilia is among the most hideous behaviors condemned by society, a more comprehensive and transdisciplinary approach is recommended in court.

## Introduction

In the last two decades, new insights on the biological bases of human behavior have led to a notable change in how social phenomena are interpreted. One important example is the biological study of sexual arousal (e.g., Schober and Pfaff, 2007; Brunetti et al., 2008), which, from an evolutionary prospective, is considered the basis of social interaction (Toates, 2009). On one hand, the biological and cognitive processes that determine sexual arousal have been better understood, and biological (Poeppl et al., 2016) and cognitive (Stoléru et al., 2012; Mohnke et al., 2014) models of sexual arousal have been formalized. On the other hand, the experimental method is increasingly applied to the study of sexuality, mainly when altered sexual arousal is observed (Mohnke et al., 2014; Sartori et al., 2016).

An interesting topic for which this transdisciplinary approach is increasingly important is the assessment of pedophilia (Seto et al., 1999). In the fifth edition of the Diagnostic and Statistical Manual of Mental Disorders (DSM 5, American Psychiatric Association, 2013), pedophilia has been depathologised, as it is defined as a mere sexual preference for prepubescent children. Importantly, the DSM 5 clearly differentiates pedophilia from paedophilic disorder, which also requires the acting out of sexual preferences on a behavioral level. Pedophilia is characterized by extensive structural and functional brain alterations (for reviews see Mohnke et al., 2014; Tenbergen et al., 2015) that do not influence the individual's ability to do otherwise (Tenbergen et al., 2015). Thus, usually pedophiles are considered liable for their paedophilic behavior. Different is the case where pedophilia emerges late in life (Mohnke et al., 2014; Sartori et al., 2016), also called “acquired paedophilic behavior.” Twenty years ago, pedophilia emerging in senescence was referred to as “regressed pedophilia” (Cormier et al., 1995) and the paedophilic episode was considered “an opportunity for senescent pedophiles to resolve some issues underlying their paedophilic interests” (Cormier et al., 1995). Furthermore, these cases have also been previously discussed as a behavioral manifestation of pre-existent latent pedophilic urges due to impulse disinhibition (Mohnke et al., 2014).

Although some psychiatrists still consider these “psycho-dynamic” interpretations of pedophilia emerging late in life as valid (Scarpazza et al., 2018 for a critical discussion; Cohen et al., 2002; Drapeau et al., 2008), more recent neuroscientific evidences have suggested that late-onset pedophilia is likely to emerge as a consequence of brain disorders (see Mohnke et al., 2014 for a review). This has dramatic consequences on the legal phenomenon of late-onset pedophilia: even though the psycho-dynamic interpretation of the paedophilic behavior indicates that the defendant should be considered fully responsible for his own action, the neuroscientific interpretation, if proven, implicates that the defendant should be considered not guilty by reason of insanity (Gilbert and Focquaert, 2015). Indeed, whether patients with acquired paedophilic behavior experienced paedophilic sexual preferences before the brain injury is irrelevant for the legal context. What is important, according to the DSM 5, is the “*behavioral fracture*” between their overt behavior prior and after the brain disease insurgence. Therefore, the law should consider the advances in scientific knowledge when dealing with mental insanity assessments. Indeed, updated scientific knowledge might be helpful to disentangle whether a *behavioral fracture* occurred and might highlight its possible causes.

In the current paper, two cases of late onset paedophilic disorder (Seto et al., 1999) are described. The reason why we believe it is important to share these data is mainly twofold. First, the existence of acquired pedophilia is still matter of debate in court, where there is need for strong scientific evidence supporting mental insanity claims. The rarity of the cases of acquired pedophilia reported in literature has already been used by prosecutors to support the idea that the existence of acquired pedophilia is still an experimental hypothesis within the scientific community (Scarpazza et al., 2018). The presence of a brain disorder to explain the late insurgence of pedophilia would, therefore, be avoided in court to support the mental insanity of the defendant (see Scarpazza et al., 2018 for a critical discussion on this topic; Farisco and Petrini, 2014). Thus, it is increasingly important to continue to provide evidences for the existence of such an uncommon condition. Second, we would like to emphasize the advantage of using the experimental method for the study of the phenomena of sexual alteration. Experimental methods rely on the existence of cognitive models for the phenomena under study, the integration of insights offered by different disciplines and the application of a variety of tools and approaches that follow the “convergence of evidence” principle (i.e., to provide evidences from different disciplines that consistently and coherently support a claim), which could be safely used in court to support a mental insanity claim (Sartori et al., 2011, 2016; Scarpazza et al., 2018). In the following, we describe how the use of the neuroscientific methods could become useful for reducing uncertainty in mental insanity assessments.

## Case 1

A 70 plus-year-old retired tradesman was reported to the authorities for enacting sexually-inappropriate behavior toward a male adolescent. In particular, he approached the boy by talking about sports, and then he touched his penis and testicles. He was jailed for 1 year and 2 months for his paedophilic behavior. One year after the release, he was again reported to the authorities for enacting sexually-inappropriate behavior toward a boy in a cinema; in particular, the man approached the boy by talking about the movie, he then touched the boy's hand and soon after, he started to touch the boy's penis and testicles. On both occasions, paedophilic urges were carried out in a disorganized and risky manner. For instance, the defendant approached the boys in a public and crowed place with a high probability of being discovered, denoting an impulse dyscontrol rather than a planned behavior. Upon arrest, he was completely unaware of the severity, of the social and moral disvalue and of the legal implication of his behavior.

The defendant had been married for over 40 years and had three children, who described him as responsible, loving and morally impeccable. History was negative for relevant medical conditions. However, both psychiatric and neurological diagnoses were present, as 15 years earlier he presented with major depression triggered by his retirement and for which he was treated with anti-dopaminergic drugs. When, some years later, the defendant showed tremor in his hands, the psychiatrist was not alerted since he suspected a tardive dyskinesia caused by the anti-dopaminergic drug. The MRI was positive for bilateral frontal lobe atrophy. A neurological examination performed shortly after the first charge revealed a severe deficit in critical thinking, abstract thinking (e.g., he was not able to describe the difference between a child and a dwarf) and logorrhea (an indirect index of disinhibition), suggestive of dysexecutive syndrome. Accordingly, the MRI confirmed the bilateral frontal lobe atrophy. The presence of dysexecutive syndrome was further supported by the neuropsychological evaluation, which revealed that despite the fact that his general cognition and memory were within the normal range, he manifested a specific cognitive impairment consisting of a severe deficit in attention, behavioral control, impulse inhibition, preservative behavior and an inability to foresee the consequences of his own actions. His family also reported that the defendant's behavior steadily changed over the last few years. For instance, he had become obsessed with hygiene and cosmetics, he started to dress up eccentrically and he manifested affective indifference. A diagnosis of possible behavioral variant of frontotemporal dementia was thus performed based on the patient's cognitive and behavioral profile of impairment in accordance with recent diagnostic criteria (Rascovsky et al., 2011).

This clinical picture was further confirmed during the forensic psychiatric evaluation performed after the second charge, aiming to define the mental state of the defendant at the time of the sexual crimes. In particular, the cognitive profile of the defendant was worsened as reflected by the decline in daily living autonomy, which is an important aspect for the diagnosis and progression of dementia, and especially of worsening of the behavioral profile. Indeed, he was unable to understand the value of money, he acted irrationally (e.g., bought expired food), he manifested obsessive behaviors (e.g., he labeled every object in the house), he become verbally aggressive toward his wife, he manifested scarce empathy, hyperphagia and hypersexuality toward his wife, he spent all his savings and he manifested impulsive behavior with kleptomania (e.g., he stole umbrellas from shops).

According to the judge's expert, the behavioral variant of frontotemporal dementia could well account for all the observed symptoms and signs, both legally irrelevant (e.g., emotional indifference, inability to understand the value of money) and legally relevant (e.g., impulse dyscontrol, hypersexuality). Furthermore, the frontotemporal dementia also proved to be fundamental to understanding the reason why this man started to change when he was more than 70 years old, to explain this sort of “behavioral fracture,” from being loving and responsible to an individual with overt (at least verbally) aggressive behavior, overt hypersexuality and socially-inappropriate behaviors. The progressive involvement of the frontal lobe is clearly in anatomo-clinical correlation with both the steadily progressive frontal dysexecutive syndrome (which mainly consists of deficits in planning, abstract thinking, flexibility and behavioral control; Godefroy et al., 2010) that emerged from the longitudinal evaluations and is consistent with the behavioral changes reported and observed (Devenney et al., 2015).

## Case 2

A 60 plus-year-old male was reported to the authorities for enacting sexually-inappropriate behavior toward a male child in front of a primary school garden. In particular, the man forced the child to touch his penis and urinated on the child's hands. After his arrest, many children declared that the man was seen masturbating close to the school fence. Similar to Case 1, paedophilic urges were carried out in a disorganized and risky manner: the defendant approached the children in a public and crowded place, with a high probability to be discovered. Furthermore, one of the children he approached lived in his neighborhood, thus heightening the probability of being correctly identified, as happened. The children's parents subsequently clarified that yes, the man was repeatedly seen masturbating close to the school fence in the last year and a half, but never before.

The defendant had been married for over 30 years and had two children. History was negative for relevant medical and neurological disorders, but positive for psychiatric disorders: the defendant suffered from clinically relevant major depression for the past 30 years. He was actually treated with delorazepam, which caused him severe asthenia and apathy. For this reason, the defendant was unemployed for years. History was also positive for slight sexually-inappropriate behaviors: when he was enlisted in mandatory military service, he was known to address women with comments with sexual connotations, for which he was reported to the military authorities. According to the documentation available, the harassments were limited to verbal comments and never reached a physical approach.

One year after the crime he was charged with, he showed clear episodes of spatiotemporal disorientation. For this reason, a CT scan was requested. The CT scan revealed the presence of a bulky meningioma, whose location at the level of the frontal and parietal right lobes caused a significant mass effect with lateral ventricles compression and inter-hemispheric shifting of the midline structures. According to the neuroradiological report, the meningioma caused direct and indirect damage to the entire brain. Given the site and dimension of the meningioma, and because of the clinical symptomatology that emerged, the defendant underwent a surgical resection of the tumor 1 month later. The histopathological examination determined that the tumor was a meningothelial meningioma.

Due to this new evidence, the court requested a psychiatric evaluation to understand the defendant's mental state when he committed the crimes. Thus, the psychiatric evaluation was performed after the surgical resection of the tumor, in particular, 9 months after the tumor resection. At the forensic psychiatric examination, no psychotic symptoms were evident, although he appeared fatuous and avoidant. He realized the moral disvalue of his behavior. The spontaneous verbal production was poor. Despite the fact that the defendant reported a clear improvement in his cognitive functions after the resection of the tumor, the neuropsychological evaluation underlined a severe cognitive impairment characterized by a general cognition below the normal range, constructional apraxia, impaired sustained attention, a clear difficulty to inhibit the automatic answer and behavior and an impairment in problem solving and planning abilities. Qualitatively, the performance of the defendant at the tests measuring these cognitive functions was characterized by the presence of perseverative answers and an inability to follow the test instructions/rules.

Meningothelial meningiomas are slow growing, *per se* benign tumors, but they become dangerous as their size increases since they compress the surrounding brain structures, causing indirect damage. According to the judge's expert, because of its dimension and location, the meningioma could well account for all the observed symptoms and signs, both legally irrelevant (e.g., spatiotemporal disorientation due to parietal lobe dysfunction) and legally relevant (e.g., impulse dyscontrol due to frontal lobe dysfunction). Indeed, the timeline of crime-related behavior is of a critical importance: there was a progression from the absence of child molestation behaviors to the insurgence of these sexually-inappropriate behaviors when the defendant was 61-years-old, 2 years before the meningioma was discovered. This temporal specificity is consistent with the statement of the children and their parents. Altogether, these data suggest that the defendant's sexual deviancy, which began approximately 1 year before his arrest, coincided with the achievement of critical mass of the tumor volume in his brain. Moreover, at the time of the MRI, the meningioma was large enough to compress the right frontoparietal lobes and to produce a significant mass effect with inter-hemispheric shifting of the midline structures. The involvement of the frontal lobe is clearly in anatomo-clinical correlation with the dysexecutive syndrome that emerged at the neuropsychological evaluation (Godefroy, 2003), persisting after the surgical resection of the tumor and, consequently, present at the time of the crime-related behaviors.

## Discussion

Acquired pedophilia has already been described as an emergent symptom of a tumor directly involving the frontal lobe (Regestein and Reich, 1978; Burns and Swerdlow, 2003; Meynen, 2016) or indirectly (Sartori et al., 2016) or as a symptom of frontotemporal dementia (Mendez and Shapira, 2011−2 cases-; Rainero et al., 2011), this latter being known to predispose individuals to criminal violation (Mendez, 2010). Importantly, despite the variability of the etiology underlying the pedophilia insurgence, all these cases have in common the pathological involvement of the prefrontal cortex.

In the current review, particular emphasis was placed on the logical reasoning supporting the conclusions that both senescent pedophiles should be considered not guilty by reason of insanity. In particular, we would like to focus attention on the interpretation within the neurophenomenological model of sexual arousal (Stoléru et al., 2012; Mohnke et al., 2014) of the symptoms experienced by the two men. According to this model, sexual arousal is comprised of three main components: cognitive, emotional/autonomic and control inhibition components. Interestingly, one or more of these components are impaired in the described cases.

In the first case, all three components are impaired: the cognitive component is impaired as the man did not understand the social and moral disvalue of his behavior, he manifested perseverative behaviors and he was unable to foresee the consequences of his own actions; and the emotional/autonomic component is impaired, as he manifested an affective indifference and lack of empathy. These cognitive and affective abilities are highly important for the capacity of self-determination. How could a person who lacks empathy understand the emotional signals coming from the victims of his aggression? How can an individual incapable of understanding moral violations evaluate the moral implications of his own actions so that he may inhibit them before they are carried out? In addition, the first man also showed an impaired control inhibition component, as he acted in a risky manner, he manifested impulsivity and disinhibition both in daily life (logorrhea, kleptomania) and at the neuropsychological evaluation.

The second case is more difficult since at the time of the mental insanity assessment, the brain tumor had been already removed. For instance, the man was able to understand the severity of his behavior, but we do not know if this was true also when he committed the crimes. Critically, despite the resection of the tumor, he still manifested impulsive behaviors, as revealed by the neuropsychological assessment. The lack of control inhibition is a critical component of the model of sexual arousal and, importantly, is what, according to the DSM 5, differentiates pedophilia (not legally prosecutable) from paedophilic behavior (legally prosecutable). Furthermore, in this second patient, the temporal specificity of symptoms insurgence is critically important, as it supports the idea that the symptoms emerged when the meningioma achieved a critical mass.

Considering the symptomatology described in the two patients, do these men have culpable mental states (*mens rea*) at the time of their acts? Are they responsible agents? In this paper, we provided a strict logical reasoning to support the idea that the answer to this question is no, they could not be considered responsible agents. Indeed, these two men should be considered not liable by reason of insanity because they lacked the capability to adapt their own behavior to social situations and to modulate the sexual impulses they were experiencing. In legal terms, they lack the capability to do otherwise (to will). However, in these cases, the paedophilic disorder emerged as a direct consequence of a brain disease. Thus, while the criteria to determine legal liability are behavioral, as previously suggested (Sartori et al., 2011), neuroscience may be a potential tool to understand the causal link between a pathological mental state and criminal behavior. It is worth stressing that these men were not considered not responsible because they suffer from frontotemporal dementia and meningioma, respectively, but because they showed a severe and erratic symptomatology that affected their so-called “free will” (Lavazza, 2016). The criteria for responsibility are, thus, symptoms-based and not diagnosis-based.

## Conclusion

The use of a transdisciplinary, scientifically-grounded approach can help to change the way legal phenomenon is interpreted. In particular, when assessing mental insanity, consultants should not only investigate the eventual existence of a diagnosis, but should assess the cognitive/affective abilities that are necessary to understand our own behavior and emotions as well as those of others. However, so far, a gold standard for the cognitive/affective assessment does not exist, limiting transdisciplinary communication. It is worth emphasizing that the use of this neuroscientific logic, which is based on updated scientific evidences and refers to cognitive/biological/behavioral models of normal function, can change the legal concept of responsibility or culpability. Given that pedophilia is among the most hideous behaviors condemned by society, a more comprehensive and transdisciplinary approach is recommended in court.

## Ethics statement

Informed consent was not obtained since we extracted the reported information by the anonymized ruling that, according with the Italian law are publicly available. Furthermore, we removed from the case descriptions all the details that could lead to patients identification.

## Author contributions

AP observed both the patients in the role of expert for the judge. CS and GS wrote the manuscript. AP critically revised the manuscript. All the authors approved the final version of the manuscript for submission.

### Conflict of interest statement

The authors declare that the research was conducted in the absence of any commercial or financial relationships that could be construed as a potential conflict of interest.
